# A new workflow of the on-line 1.5-T MR-guided adaptive radiation therapy

**DOI:** 10.1007/s11604-023-01457-4

**Published:** 2023-06-24

**Authors:** Takashi Uno, Masato Tsuneda, Kota Abe, Yukio Fujita, Rintaro Harada, Makoto Saito, Aki Kanazawa, Asuka Kodate, Yukinao Abe, Yohei Ikeda, Miho Watanabe Nemoto, Hajime Yokota

**Affiliations:** 1 Diagnostic Radiology and Radiation Oncology, Graduate School of Medicine, Chiba University, Inohana 1-8-1, Chuou-ku, Chiba City, Chiba 260-8670 Japan; 2 Department of Radiation Oncology, MR Linac ART Division, Graduate School of Medicine, Chiba University, Inohana 1-8-1, Chuou-ku, Chiba City, Chiba 260-8670 Japan; 3 Department of Radiology, Chiba University Hospital, Inohana 1-8-1, Chuou-ku, Chiba City, Chiba 260-8670 Japan

**Keywords:** MRgART, MR-Linac, Workflow, Adaptive radiation therapy, Adapt to shape

## Abstract

**Purpose:**

The aim of this study was to develop a new workflow for 1.5-T magnetic resonance (MR)-guided on-line adaptive radiation therapy (MRgART) and assess its feasibility in achieving dose constraints.

**Materials and methods:**

We retrospectively evaluated the clinical data of patients who underwent on-line adaptive radiation therapy using a 1.5-T MR linear accelerator (MR-Linac). The workflow in MRgART was established by reviewing the disease site, number of fractions, and re-planning procedures. Five cases of prostate cancer were selected to evaluate the feasibility of the new workflow with respect to achieving dose constraints.

**Results:**

Between December 2021 and September 2022, 50 consecutive patients underwent MRgART using a 1.5-T MR-Linac. Of these, 20 had prostate cancer, 10 had hepatocellular carcinoma, 6 had pancreatic cancer, 5 had lymph node oligo-metastasis, 3 had renal cancer, 3 had bone metastasis, 2 had liver metastasis from colon cancer, and 1 had a mediastinal tumor. Among a total of 247 fractions, 235 (95%) were adapt-to-shape (ATS)-based re-planning. The median ATS re-planning time in all 50 cases was 17 min. In the feasibility study, all dose constraint sets were met in all 5 patients by ATS re-planning. Conversely, a total of 14 dose constraints in 5 patients could not be achieved by virtual plan without using adaptive re-planning. These dose constraints included the minimum dose received by the highest irradiated volume of 1 cc in the planning target volume and the maximum dose of the rectal/bladder wall.

**Conclusion:**

A new workflow of 1.5-T MRgART was established and found to be feasible. Our evaluation of the dose constraint achievement demonstrated the effectiveness of the workflow.

## Introduction

In December of 2021, radiation therapy utilizing the 1.5-T MR-Linac system (Elekta AB, Stockholm, Sweden) was initiated. This innovative system merges a 7MV flattening filter free (FFF) Elekta linear accelerator with a Philips 1.5-T MRI scanner (Philips Healthcare, Amsterdam, the Netherlands) [1–4]. This cutting-edge MR image-guided radiation therapy (IGRT) allows for a timely and appropriate treatment re-planning to be conducted while the patient is situated on the treatment couch [5, 6]. This facilitates an immediate adaptive radiotherapy approach based on both the position of the tumor and normal tissue during daily treatment, using near-real-time beam-on cine MR imaging during treatment. This procedure, termed MR-guided on-line adaptive radiation therapy (on-line MRgART), necessitates meticulous off-line treatment planning and a seamless on-line process on the day of the treatment. However, the actual on-line MRgART method varies across institutions, with no standardized methodology having been established thus far [7–14]. In this paper, we present the establishment of a novel workflow for on-line MRgART at our institution, as well as an evaluation of the feasibility of this new workflow in regard to meeting dose constraints.

## Materials and methods

**Fig. 1:**
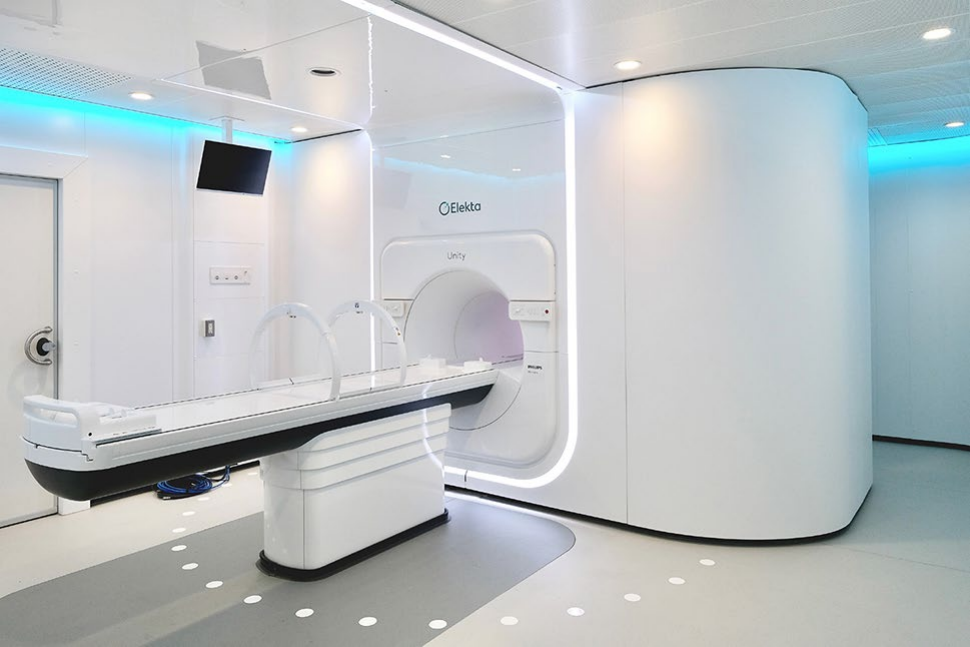
Elekta Unity 1.5-T MR-Linac system. The internal structure consists of a linear accelerator rotating gantry placed outside of the MRI magnets

On-line MRgART using Elekta Unity (Fig. 1) was initiated in December 2021. The basic concept and structure of this treatment machine has been described by Raaymakers et al. [4]. The system is composed of a 1.5-T MRI magnet, surrounded by a large rotating gantry of linear accelerators, on which a magnetron, a tri-pole electron gun, and an S-band standing wave accelerating tube are located. The magnetic field strength of the donut-shaped space containing the electron gun is designed to be almost zero while maintaining the uniformity of the main magnetic field so that electrons emitted from the gun reach the acceleration tube. The therapeutic beam is delivered through a slit in the center of the magnet. Basic specifications are outlined in Table 1. The 1.5-T MR-Linac allows for MR imaging in off-line treatment planning, on-line treatment planning, and after treatment irradiation completion. Near-real-time two-dimensional cine MR images at 5 frames per second can be obtained in three cross sections prior to and during the treatment beam delivery, also known as beam-on imaging.

**Table 1:** Basic specification of Elekta Unity 1.5-T MR-Linac system

Strength of static magnetic field	1.5T
Magnetic field stability	≦ 0.1 ppm/h
Peak amplitude	34 mT/m
Peak slew rate	120 T/m/s
Imaging sequences	T1WI, T2WI, FLAIR, DWI, Cine bFFE
Bore size (diameter x depth)	70 × 132 cm
Beam energy	7 MV, Flattening Filter Free
Source to axis distance	143.5 cm
Maximum field size (head–tail × lateral)	22 × 57.4 cm
Dose rate	425 MU/min
Gantry rotation speed	6 RPM
Multi-leaf collimator	160 (80 pairs)
Max leaf travel per second	60 mm

T1WI, T1-weighted imaging; T2WI, T2-weighted imaging; FLAIR, fluid-attenuated inversion recovery; DWI, diffusion-weighted imaging; bFFE, balanced fast field echo

### Workflow of the adaptive radiation therapy

**Fig. 2:**
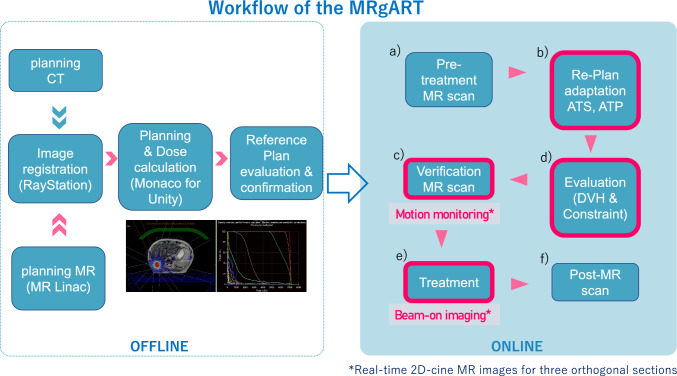
Workflow of the MR-guided on-line adaptive radiation therapy (MRgART). The left side of the figure shows the off-line procedure of creating an MR-Linac reference plan prior to the treatment start day. On-line workflow of the adaptive planning was depicted on the right half. Some of the key steps of the on-line MRgART are circled in red. **a** Pre-treatment MR imaging with the MR-Linac system. **b** Re-planning with modification of targets, risk organs, or body surface contours (ATS: adapt to shape) and re-planning without any contour modification (ATP: adapt to position). **c** Evaluation of dose distribution and DVH and assessing achievement status of dose constraints. **d** Reconfirm the location of the tumor and risk organs with MRI just prior to irradiation. **e** Irradiation while confirming beam-on imaging with real-time cine MR. **f** MR images are also taken immediately after the end of irradiation for confirmation

The overall workflow consists of two procedures: off-line and on-line, as shown in Fig. 2.

#### Off-line treatment planning

Patients undergo treatment planning computed tomography (CT) scan followed by MR simulation using the Elekta Unity MR-Linac system. Image registration of CT and MR is performed on the RayStation (Raysearch Laboratories AB, Stockholm, Sweden) to delineate the patient’s anatomy. Anatomical structures, including tumor and risk organs with electron density information, are transferred to Monaco for Unity (Elekta AB, Stockholm, Sweden) to create the MR-Linac reference plan before the start of treatment. Patient-specific quality assurance measurements are performed using ArcCheck (Sun Nuclear, Melbourne, United States) to confirm the acceptability of the MR-Linac reference plan.

#### On-line adaptive treatment planning

On the day of radiation therapy, pre-treatment MR images are taken using the MR-Linac system, and differences between the reference plan’s MR images and pre-treatment MR images are evaluated. Anatomical structures, such as contours of the external body, tumor, and risk organs, are modified if necessary, using deformable image registration (DIR) with or without manual adjustment. Re-planning is conducted with the patient on the treatment couch, based on the prescription to the tumor and dose constraints to the normal tissues according to the changes in contour shapes. This is called the Adapt to Shape (ATS) procedure [5]. If the contour does not need correction, and the overall position is the only misaligned component, then re-planning is conducted after only correcting the position, called the Adapt to Position (ATP) procedure. After confirming the dose–volume histogram (DVH) based on the new treatment plan, a verification MRI is obtained just before the start of treatment to ensure the contours of the target and risk organs do not require further modification. Independent dose verification using MU2net (DOSIsoft, Cachan, France) is performed by medical physicists [15]. Two-dimensional (2D) cine images in three cross sections are acquired for motion monitoring to ensure proper irradiation of the target. Irradiation is performed while continuously monitoring the 2D cine images (Beam-on monitoring). After treatment completion, another MRI is taken to confirm the position of the tumor and risk organs.

### Personnel and staff

The institution has a team of five radiation oncologists who are board-certified, four full-time medical physicists, seven full-time radiation therapy technologists, and three nurses who are all involved in radiation therapy and patient care. For MR-Linac treatments, at least one radiation oncologist, one medical physicist, one technician, and one nurse are present as a unit. Additionally, an expert MR technician operates the MRI machine off-line and on-line to ensure imaging is effective and safe.

### Feasibility of the ATS re-planning

The feasibility of the ATS re-planning was assessed by selecting five cases of prostate cancer with less respiratory movement in both the target and risk organs compared to abdominal tumors like liver or pancreatic cancer. A total of five cases were selected for feasibility studies, one for every three or four cases, to avoid at least time bias. Four cases were intermediate risk, and one was high risk. Absorbable SpaceOAR Hydrogel (Boston Scientific, *Marlborough, United States*) was implanted in all five cases. The clinical target volume included the prostate and the entire seminal vesicle in one case, and the prostate and the proximal 1 cm of the seminal vesicle in the other four cases. The planning target volume was created by expanding the clinical target volume by 5 mm except posteriorly, where a 3 mm margin was used. Treatment plans were created for these five cases with both actual ATS (Plan 1) and a virtual plan (Plan 2) by fixing the segments and fluences of the reference plan while only changing the irradiation positions after contour modification. The dose constraint achievement of these two plans was compared in each case. Dose constraints to be met were set with reference to reported data, including the minimum dose received by the highest irradiated volume of 1 cc (D1cc) of the planning target volume (PTV) and the maximum dose (Dmax) of the rectal/bladder wall (Table 2) [16].

**Table 2:** Dose constraints for prostate cancer irradiated in 5 fractions using MR-Linac

Definition	Constraints (acceptable)
PTV	
D98	> 36.0 Gy (96%)
D95	> 37.5 Gy (100% = prescription)
D1cc	< 39.4 Gy (105%)
Rectal wall	
Dmax	< 38.6 Gy (103%)
D1cc	< 38.5 Gy
D53	24 Gy
Rectum	
V30.15 Gy	< 8 cc
Bladder wall	
Dmax	< 105%
D1cc	42 Gy
D53	24 Gy

PTV, planning target volume; Dxx, the minimum dose received by XX% volume; Dmax, the maximum dose; D1cc, the minimum dose received by the highest irradiated volume of 1 cc

## Results

At the end of September 2022, a total of 50 patients consecutively received MRgART using this treatment procedure. The number of radiation fractions administered to each patient varied between 2 and 8, with a median of 5. Ninety percent of the patients (45 out of 50) were treated in 5 fractions. The patients had various types of cancer, including prostate cancer (20 patients), hepatocellular carcinoma (10 patients), pancreatic cancer (6 patients), lymph node oligo-metastasis (5 patients), renal cancer (3 patients), bone metastasis (3 patients), liver metastasis from colon cancer (2 patients), and mediastinal tumor (1 patient).

Out of a total of 247 fractions administered to the 50 patients, most of the fractions (95%) involved adaptive re-planning using ATS procedure, with only 12 fractions using ATP procedure. For all patients, the time required from pre-treatment MR image acquisition to the start of actual irradiation after on-line adaptive re-planning in total fractionated irradiations was recorded. The median of the average time for ATS re-planning in all 50 cases was 17 min, ranging from 3 to 58 min. For each disease site with three or more cases, the median time required for ATS re-planning was 46 min for pancreatic cancer, 18 min for hepatocellular carcinoma, 17 min for prostate cancer, 14 min for lymph node oligo-metastasis, 17 min for renal cancer, and 7 min for bone metastasis (Table 3). On the other hand, the median time required for re-planning by ATP was 2 min, with a range of 1 to 7 min.

**Table 3:** Average time required for adapt to shape (ATS) re-planning for disease site with three or more cases (n = 47)

Treatment site (*n*)	Average time required for re-planning (min)	
	Range	Median
Prostate cancer (20)	11–29	17
Hepatocellular carcinoma (10)	9–34	18
Pancreatic cancer (6)	25–58	46
Lymph node oligo-metastasis (5)	12–19	14
Renal cancer (3)	12–18	17
Bone metastasis (3)	3–22	7

Of all 247 fractions, there were interruptions in a total of 25 on-line MRgART procedures; patients had to interrupt their treatment due to urination or other discomfort in 7 fractions, and patients had to get off the couch due to significant changes in organ position in 15 fractions. Also, in three fractions, the treatment was interrupted due to the troubles with the equipment. However, finally, the treatment was completed in all fractions.

**Fig. 3:**
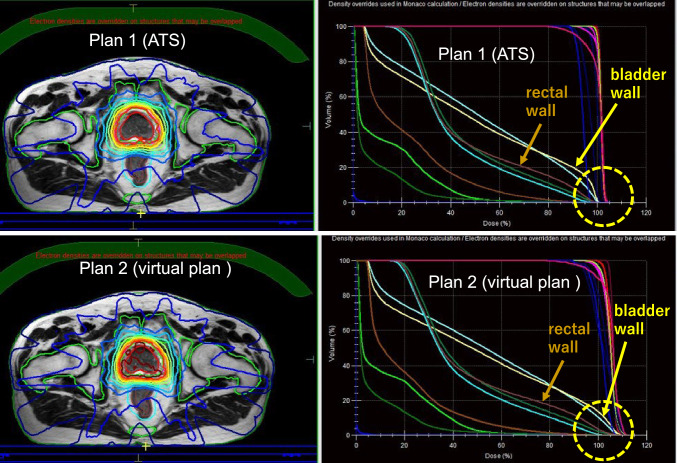
Comparison between the dose distribution and the dose-volume histogram in actual ATS plan and a virtual plan in patient with prostate cancer. All dose constraints are met in the actual ATS (Plan 1), but neither the minimum dose received by the highest irradiated volume of 1 cc (D1cc) in the planning target (PTV) volume nor the maximum dose (Dmax) in the rectal/bladder wall are achieved in the virtual plan (Plan 2). In the virtual plan, both Dmax of the rectal and bladder wall are nearly 110% of the prescribed dose (yellow dotted circles)

In the feasibility assessment of dose constraints in five prostate cancer patients, all dose constraints were successfully met in all 5 patients using ATS-based re-planning (Plan 1). In contrast, a total of 14 dose constraints among the 5 patients could not be met using Plan 2, in which only the irradiation position was adjusted after contour modification (Table 4). The main dose constraints that could not be met were D1cc of the PTV, Dmax of the rectal wall, and Dmax of the bladder wall. Figure 3 shows a case in which the dose constraints of the rectum wall and bladder wall could not be met with a virtual plan (Plan 2) but could be met with an ATS-based adaptive re-planning.

**Table 4:** Dose constraints that could not be met by re-planning only after modification of irradiation position without performing adapt-to-shape (ATS) procedure

Dose constraints	*N* (cases)
D1cc of the PTV (< 105%)	5
Dmax of the bladder wall (< 105%)	5
Dmax of the rectal wall (< 103%)	4

Prescribed dose for PTV was 37.5 Gy in 5 fractions

Abbreviations are the same as in Table 2

## Discussion

On-line MRgART is a novel technique that employs near-real-time MRI to precisely guide the administration of radiation therapy. This state-of-the-art approach provides visualization of tumors and surrounding normal tissue during radiation treatment, thereby enhancing treatment accuracy and minimizing damage to normal tissue. Compared to traditional CT-based image-guided radiation therapy, the benefits of 1.5-T MRgART are numerous. First, radiation therapy is delivered with superior accuracy owing to the high contrast resolution of 1.5-T MR images, which provide detailed information about the location, size, and shape of the tumor. Second, MRgART enables near-real-time visualization of tumors and surrounding tissue during radiation treatment, thereby allowing for the direct monitoring, capture, and control of irradiation without the need for additional radiation exposure or invasive insertion of metal markers for image guidance. This allows for immediate adjustments to the radiation delivery, ensuring that the tumor receives the maximum dose while minimizing exposure to normal tissue. Finally, adaptive re-planning based on MR images immediately prior to irradiation can recreate optimal dose distributions based on the patient's individual anatomy and response to treatment [17–19]. These advantages are expected to result in more precise dose delivery to the target, reduced radiation exposure of risk organs, and improved DVH parameters. This is particularly relevant for tumors located near critical organs or structures. Online MRgART thus expands and promotes the use of increased fractional doses, fewer fractionations, and hypo-fractionated irradiation in the treatment of various types of cancer. For pancreatic cancer, prostate cancer, lymph node recurrence, etc., excluding brain tumors and/or head and neck tumors, the number of fractions is typically five, according to the 2020 Consortium Meeting [18].

The Monaco for Unity treatment planning device has two key functions: ATP procedure and ATS procedure. In ATP, the MR image obtained immediately before treatment is matched with the planned MR image via rigid registration, the shift value of the irradiated area is calculated, planning and optimization are performed again, and dosimetry of the target and risk organs are evaluated. In ATS, the contour shape is compared between the MR image obtained immediately before treatment and the treatment planning MR image, and then re-planning is performed after correction of anatomical shapes. The contour information of the tumor and normal tissue depicted in the MR image obtained immediately before treatment is newly adopted, and planning and optimization are performed again using the electron density obtained from CT for each contour. While ATP is a re-planning process that primarily corrects the position, ATS constitutes a complete re-planning process that utilizes the new contour information, and therefore requires more time [5, 11, 13, 14].

We have implemented an institutional workflow for on-line MRgART. We deem this approach feasible because of its adherence to dose constraints, as demonstrated in the feasibility study. MR-based IGRT allows accurate delineation of tumor and risk organs due to its superior contrast resolution compared to CT. Certainly, re-planning using MR images taken just prior to treatment is more time-consuming and labor-intensive than CT-based radiotherapy. However, this can be mitigated by pre-drawing the tumor and organ contours during MR simulation with the treatment device itself. This permits delineation of the contours on new MR images on the day of treatment with minor modifications based on the MR of the reference plan. In most cases, ATS was required for re-planning, although employing the deformable image registration (DIR) technique necessitated only minor manual contour corrections. Compared with other reports [11, 14], the unique feature of our workflow is that the MR-Linac itself, rather than other MRIs such as simulator or diagnostic MRIs, is used to take the treatment planning MRI and create the reference plan. The ability to use images taken with the same bed and coils on the same device to compare a patient's anatomy between pre-treatment MR images and those of the reference plan is significant. It reduces the time required for contour correction and is expected to have minimal effect on DVH and other parameters due to the good consistency of MR images. The time required for modifying organ contours was shorter for liver cancer, renal cancer, and lymph node oligo-metastasis due to the limited number of gastrointestinal tracts bordering the tumor. However, the time required for on-line re-planning for pancreatic cancer was relatively longer as the pancreas is surrounded on three sides by the stomach, small intestine, and large intestine.

The workflow for on-line MRgART we have established seems to contribute to the improved throughput of the daily treatment procedures owing to the effective cooperation of the medical team and resultantly to the learning curves of the staff members.

In conclusion, we have established a workflow for on-line MRgART using the newly implemented Elekta Unity 1.5-T MR-Linac system. The initial evaluation of the dose distribution and DVH demonstrated that the established workflow was feasible. As the clinical practice of on-line MRgART has only recently begun, we anticipate further improvements and evolution in future.
